# “Hot spots” of N and C impact nitric oxide, nitrous oxide and nitrogen gas emissions from a UK grassland soil

**DOI:** 10.1016/j.geoderma.2017.06.007

**Published:** 2017-11-01

**Authors:** Nadine Loick, Elizabeth Dixon, Diego Abalos, Antonio Vallejo, Peter Matthews, Karen McGeough, Catherine Watson, Elizabeth M. Baggs, Laura M. Cardenas

**Affiliations:** a Rothamsted Research, North Wyke, Okehampton, Devon EX20 2SB, UK; b Technical University of Madrid, Chemistry and Agricultural Analysis, Madrid, Spain; c School of Geography, Earth and Environmental Sciences, University of Plymouth, Davy Building, Drake Circus, Plymouth, Devon PL4 8AA, UK; d Agri-Food and Biosciences Institute, Newforge Lane, Belfast BT9 5PX, UK; e The Royal (Dick) School of Veterinary Studies, University of Edinburgh, Easter Bush Campus, Midlothian EH25 9RG, UK

**Keywords:** Denitrification, Flow-through system, Isotopes, Nitrogen cycle, Greenhouse gas (GHG) emissions

## Abstract

Agricultural soils are a major source of nitric- (NO) and nitrous oxide (N_2_O), which are produced and consumed by biotic and abiotic soil processes. The dominant sources of NO and N_2_O are microbial nitrification and denitrification, and emissions of NO and N_2_O generally increase after fertiliser application.

The present study investigated the impact of N-source distribution on emissions of NO and N_2_O from soil and the significance of denitrification, rather than nitrification, as a source of NO emissions. To eliminate spatial variability and changing environmental factors which impact processes and results, the experiment was conducted under highly controlled conditions. A laboratory incubation system (DENIS) was used, allowing simultaneous measurement of three N-gases (NO, N_2_O, N_2_) emitted from a repacked soil core, which was combined with ^15^N-enrichment isotopic techniques to determine the source of N emissions.

It was found that the areal distribution of N and C significantly affected the quantity and timing of gaseous emissions and ^15^N-analysis showed that N_2_O emissions resulted almost exclusively from the added amendments. Localised higher concentrations, so-called hot spots, resulted in a delay in N_2_O and N_2_ emissions causing a longer residence time of the applied N-source in the soil, therefore minimising NO emissions while at the same time being potentially advantageous for plant-uptake of nutrients. If such effects are also observed for a wider range of soils and conditions, then this will have major implications for fertiliser application protocols to minimise gaseous N emissions while maintaining fertilisation efficiency.

## Highlights

• Gaseous emissions were studied at the core scale under highly controlled conditions.
• N_2_O and N_2_ emissions from denitrification depended on applied amounts of N and C.
• NO emissions did not depend on applied N and C concentration but on surface area.
• Between 70 and 80% of N_2_O emissions derived from the applied N.
• About 30% of the amount of applied N was lost via gaseous emissions.

## Graphical abstract

**Figure:**
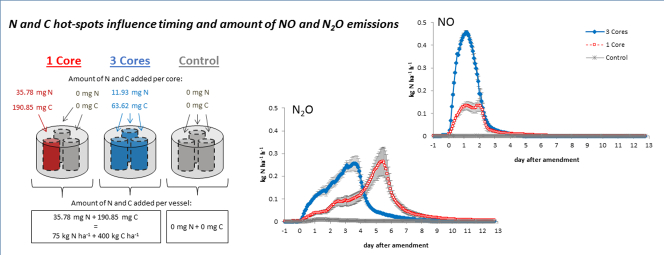

## 1 Introduction

Agricultural soils are a dominant source of nitrous oxide (N_2_O) and nitric oxide (NO) emissions (IPCC, 2007b, Ravishankara et al., 2009). N_2_O is a potent greenhouse gas (GHG) with a global warming potential 298 times that of CO_2_ for a 100-year timescale (IPCC, 2007a), while NO catalyses the formation of ground level ozone affecting human health and vegetation (Crutzen, 1981) and takes part in the formation of acid rain and the eutrophication of semi-natural ecosystems. Both gases are produced in soils by nitrification, denitrification, nitrifier denitrification and nitrate ammonification (Baggs, 2011). Which of these processes dominate in soil depends on several factors such as pH, temperature, nutrient availability, soil structure and soil water filled pore space (WFPS). Denitrification is a mainly bacterially mediated process occurring under absence/limitation of oxygen (O_2_) as most denitrifying bacteria are facultative anaerobes. In addition, most denitrifying bacteria couple nitrate (NO_3_^−^) reduction with organic carbon (C_org_) oxidation to gain energy, making a supply of readily available C_org_ a usual requirement for denitrification to occur (Knowles, 1982). High WFPS reduces the oxygen availability within the soil by replacing air in soil pores with water and with available C_org_ present, this promotes denitrification. Inhomogeneous fertiliser application or excretions of grazing animals can change the factors influencing the processes resulting in high NO and N_2_O emissions in small areas, creating hot-spots of microbial activity.

In a comprehensive review Saggar et al. (2013) described the biological and chemical characteristics of denitrification. The denitrification process consists of several reactions with each reaction supplying the substrate for the subsequent one. Each reaction becomes progressively energetically less favourable. When the soil microbial community is supplied with NO_3_^−^ as the first substrate of denitrification, it is transformed via NO_2_^−^ to NO. NO is a very reactive gas, as well as toxic to most organisms (Richardson et al., 2009). Because of its toxicity, most organisms produce the enzyme nitric oxide reductase (Nor) which catalyses the transformation of NO to N_2_O, resulting in low NO:N_2_O ratios. During the next step in the denitrification process N_2_O is transformed by the nitrous oxide reductase (Nos) to nitrogen gas (N_2_). However, the denitrification systems of most fungi and around one third of sequenced denitrifying bacteria lack the gene encoding Nos and consequently for those organisms, N_2_O will evolve as the final denitrification product rather than N_2_ (Saggar et al., 2013), resulting in larger N_2_O:N_2_ ratios. Both NO:N_2_O and N_2_O:N_2_ ratios have been used as indicators for the relative contribution of denitrification and nitrification and the availability of C, respectively (del Prado et al., 2006, Scheer et al., 2009, Wang et al., 2011, Wang et al., 2013).

Microbial denitrification is often the dominant process generating N_2_O and there is a good understanding of the abiotic factors regulating N_2_O emissions via denitrification (Beaulieu et al., 2011). However, even though NO is an obligatory intermediate of N_2_O formation in denitrification it is quickly reduced (Wolf and Russow, 2000, Russow et al., 2009).

Most experiments suggest that NO emitted from soils is mainly produced through nitrification (Skiba et al., 1997). Under denitrifying conditions, favoured by high water content, soil compaction and fine soil texture, there is consequently a low diffusivity, so it has been assumed that NO is further reduced to N_2_O before it escapes to the soil surface (Skiba et al., 1997). Recent findings, however, challenge these assumptions (Loick et al., 2016). Using the gas-flow-soil-core technique which has been proven to be a reliable tool for quantifying emissions from denitrification, Wang et al. (2013) observed significant NO fluxes from NO_3_^−^-amended soils. Attributing these emissions specifically to denitrification has previously remained elusive due to methodological constraints, which used to rely on acetylene inhibition and isotope labelling techniques but with no ability to directly quantify ^15^N-NO production (Baggs, 2008).

One factor affecting denitrification is the amount of N available to the denitrifying microbial community. It has been shown that with increasing NO_3_^−^ concentrations, the positive relationship between NO_3_^−^ concentrations and denitrification rates (NO_3_^−^-N < 1 mmol (Ogilvie et al., 1997, Zhong et al., 2010)) changes to a negative one when NO_3_^−^-N concentrations are above 50 μg g^− 1^ soil (Luo et al., 1996) or from 2 to 20 mM (Senbayram et al., 2012). On grazed fields, N is deposited at very high but localised concentrations via livestock excreta. The high concentration of N and available C in urine and dung result in a relatively high default emission factor of 2% of the applied N, but emissions also vary with pH and salinity (van Groenigen et al., 2005). Although applying fertiliser to grass- or arable land via spreaders distributes the N more evenly, there are still ‘hot spots’ of N around fertiliser granules. There is still large uncertainty about the contribution of these hot-spots to net GHG emissions. Models have been used to predict N_2_O emissions depending on soil structure (Laudone et al., 2011, Laudone et al., 2013). Understanding how hot-spots of N and C affect losses of N is crucial for the design of effective GHG mitigation strategies.

In the context of the complexity of the nitrification and denitrification processes occurring in soil, and the conflicting results which occur under varying conditions, unambiguous results can only be obtained by tightly controlling the conditions of the system and carrying out the experiments on a single soil type. The studies can then be carefully extended to other conditions and soil types, from which wider ranging conclusions can be drawn.

The aim of the present study was to investigate (i) the effects of N-source distribution on emissions of NO and N_2_O from soil under highly controlled, denitrification favouring conditions, and (ii) the significance of denitrification as a source of NO emissions. We hypothesize that nutrient concentration and application area will affect the magnitude and timing of N emissions. This would result in the need to consider different mitigation strategies depending on hot-spots of nutrient availability.

## 2 Materials and methods

### 2.1 Experimental design

To investigate the effects of nutrient concentration and application area the experimental design tightly constrained the following factors: lateral diffusion of nutrients (monitoring vertical diffusion); water filled pore space (WFPS), temperature, soil heterogeneity, surface mass transfer coefficient, ambient atmosphere (N_2_ free to measure N_2_ emissions), ratio of soil volume to nutrient concentration, and ratio of soil surface to nutrient concentration.

The implicit assumption is that we have therefore set up a one-dimensional system without any highly localised variation in WFPS and consequently without any spatial variation in microbial activity.

Conditions were chosen so that they were optimal for denitrification.

**Fig. 1:**
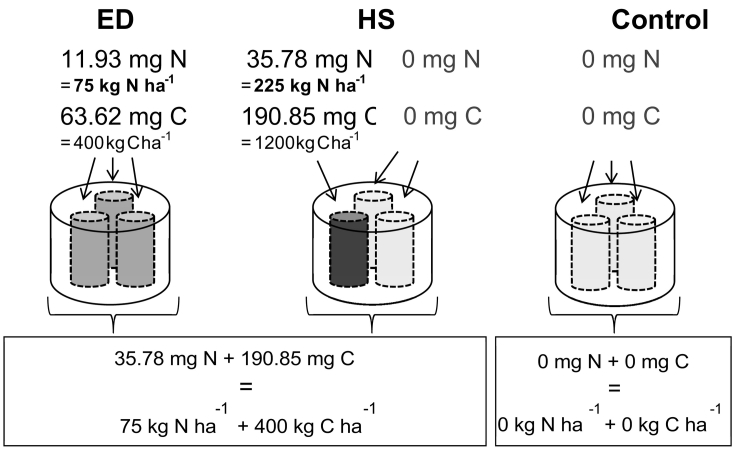
Schematic showing the treatments evenly distributed (ED), hot spot (HS) and Control and the respective amount of N and C added to each core in mg N and C (top values) and over the whole vessel (bottom numbers) in kg ha^− 1^ as well as mg per vessel. Each small core contained 95.3 g dry soil.

The incubation experiment was carried out using the DENItrification System (DENIS), a specialized gas-flow-soil-core incubation system (Cárdenas et al., 2003) in which environmental conditions can be tightly controlled. The DENIS simultaneously incubates 12 vessels containing 3 soil cores each (Fig. 1). Cores were packed to a bulk density of 0.8 g cm^− 3^ to a height of 75 mm into plastic sleeves of 45 mm diameter. To promote denitrification conditions, the soil moisture was adjusted to 85% WFPS, taking the amendment with nutrient solution into account. To measure N_2_ fluxes, the native N_2_ was removed from the soil and headspace without limiting O_2_ levels that would be present in air. This was achieved by using a mixture of He:O_2_ (80:20). First the soil cores were flushed from the bottom at a flow rate of 30 ml min^− 1^ for 14 h. To measure baseline emissions, flow rates were then decreased to 12 ml min^− 1^ and the flow re-directed over the surface of the soil core for three days before amendment application. The vessels were kept at 20°C during flushing as well as for the 13-day incubation period after amendment application.

The experiment was set up to investigate the effect of a heterogeneous distribution of N and C on gaseous emissions from denitrification, by applying a high concentration of N and C localised to only a third of the total surface area (i.e. one of the three cores) within a vessel, as opposed to an even distribution of the same amount of N and C over a three times larger area (i.e. evenly distributed over all 3 cores within a vessel). There were two reasons why the treatment was physically separated into one of three separate cores, rather than simply applying the treatment to one third of the surface of a larger core. The first was to remove subsurface lateral dispersion effects which could not be quantified. For future modelling purposes, the physical separation allows the system to be approximated as one-dimensional, to a workable level of approximation. The second reason is that gaseous emissions are controlled at the surface of the soil by the mass transfer coefficient which is directly related to the size of the transmitting layer, and diffusion through the stationary boundary layer of gas between the soil (with or without treatment) and the flowing gas stream (Laudone et al., 2011). Wetting precisely one third of the surface addressed both of these parameters.

The experiment involved the following 3 treatments (Fig. 1), with four replicate vessels per treatment: HS = hot-spot, one of the three cores inside a vessel was amended with ^15^N-KNO_3_ enriched to 5 at% and glucose; ED = equal distribution, all three of the cores inside a vessel were amended with ^15^N-KNO_3_ enriched to 5 at% and glucose; Control = only water was applied to each of the three cores. Considering the total surface area of the vessel, N was applied at a rate of 75 kg N ha^− 1^ (i.e. 125 mg N kg^− 1^ dry soil) and C as glucose at 400 kg C ha^− 1^ resulting in 35.78 mg N and 190.85 mg C per vessel. For treatment HS this resulted in all of the 35.78 mg N and 190.85 mg C being applied in solution with 5 ml water to one of the three cores, while the other two cores each received 5 ml water only. For treatment ED the same amount of N and C was diluted in 15 ml water and 5 ml of that solution were added to each one of the three cores inside one vessel. In order to maintain the incubation conditions, the amendment was applied to each of the three cores via a syringe through a sealed port on the lid of the incubation vessel.

### 2.2 Soil preparation

A clayey pelostagnogley soil of the Hallsworth series (Clayden and Hollis, 1984) (44% clay, 40% silt, 15% sand (w/w), Table 1) was collected on the 4th of November 2013 from a typical grassland in SW England, located at Rothamsted Research, North Wyke, Devon, UK (50° 46′ 50″ N, 3° 55′ 8″ W). Spade-squares (20 × 20 cm to a depth of 15 cm) of soil were taken from 12 locations along a ‘W’ line across a field of 600 m^2^ size which was surrounded by larger fields of similar grassland. After sampling, the soil was air dried to ~ 30% gravimetric moisture content, sieved to < 2 mm and stored at 4°C until preparation of the experiment. Before starting the experiment, the soil was preincubated to avoid the pulse of respiration associated with wetting dry soils (Kieft et al., 1987). For this, the required soil was spread to 3–5 cm thickness. Then, while being mixed continuously, the soil was primed by spraying it with water containing 25 kg N ha^− 1^ of KNO_3_, which is a typical yearly rate of N deposition through rainfall in the UK (Morecroft et al., 2009, RoTAP, 2012). The soil was then left for 3 days at room temperature before packing into cores and starting the incubation.

**Table 1:** Soil characteristics (before (bp) and after priming (ap) but before amendment application). Mean ± standard error (n = 3).

Parameter	Amount
pH water [1:2.5]	5.6 ± 0.27
Available magnesium (mg kg^− 1^ dry soil)	100.4 ± 4.81
Available phosphorus (mg kg^− 1^ dry soil)	10.4 ± 1.10
Available potassium (mg kg^− 1^ dry soil)	97.5 ± 12.83
Available sulphate (mg kg^− 1^ dry soil)	51.7 ± 0.62
Total N (% w/w)	0.5 ± 0.01
Total oxidised N (mg kg^− 1^ dry soil)	bp 46.0 ± 0.21
	ap 97.5 ± 0.40
Ammonium N (mg kg^− 1^ dry soil)	6.1 ± 0.09
Organic matter (% w/w)	11.7 ± 0.29

### 2.3 Gas analyses and data management

Gas samples were taken every 10 min, resulting in bi-hourly measurement for each vessel. Fluxes of N_2_O and CO_2_ were quantified using a Perkin Elmer Clarus 500 gas chromatograph (GC; Perkin Elmer Instruments, Beaconsfield, UK) equipped with an electron capture detector (ECD) for N_2_O and with a flame ionization detector (FID) and a methanizer for CO_2_. N_2_ emissions were measured by GC with a helium ionization detector (HID, VICI AG International, Schenkon, Switzerland) (Cárdenas et al., 2003), while NO concentrations were determined by chemiluminescence (Sievers NOA280i, GE Instruments, Colorado, USA). All gas concentrations were corrected for flow rate through the vessel, which was measured daily, and fluxes were calculated on a kg N or C ha^− 1^ h^− 1^ basis. CO_2_ fluxes showed constant emissions of 0.67 kg C ha^− 1^ h^− 1^ before and after the peak in all vessels. In order to show emissions attributed to amendment application only, the CO_2_ fluxes were adjusted by subtracting this baseline.

Initial emission rates for each gas and vessel were determined from the beginning of each peak until the increase in concentrations slowed down, i.e. for NO 12 h from day 0, for N_2_O 24 h from day 0, for N_2_ 36 h from day 2.5 for treatments ED and Control and from day 4.5 for treatment HS, for CO_2_ 36 h from day 0 (see Table 2).

**Table 2:** Initial production rates of measured gaseous emissions in g per hour. Mean ± standard error (n = 4). The rates were measured over the following time-periods: NO: 0–0.5 days; N_2_O: 0–1 day; N_2_: ED and Control 2.5–4 days, HS 4.5–6 days; CO_2_: 0–1.5 days. Different letters indicate significant differences between treatments (n = 4; p = 0.01). N_2_O emission rates are significantly different between ‘HS’ and ‘Control’ at the 95% confidence level (p = 0.017).

	ED	HS	Control
NO (g h^− 1^)	0.028 ± 0.001^A^	0.007 ± 0.001^B^	0.000 ± 0.00^C^
N_2_O (g h^− 1^)	4.79 ± 0.36^A^	1.55 ± 0.28^B^	0.38 ± 0.04^B^
N_2_ (g h^− 1^)	2.11 ± 1.04^A^	2.73 ± 1.52^A^	0.00 ± 0.13^A^
CO_2_ (g h^− 1^)	31.65 ± 2.48^A^	15.41 ± 1.66^B^	1.78 ± 2.23^C^

Gaseous emissions were measured per incubation vessel. Additionally emissions attributed to the amended area within a vessel were calculated (per core basis). In treatment ED and the Control all cores within a vessel received the same application, i.e. emissions calculated for the vessel are the same as when calculated for the amendment concentration. For treatment HS, however, only one core received N at a rate of 225 kg ha^− 1^. To calculate emissions from this one core only, the following equation was used:

(1) EHS∗=VHS−23VC×3

with E_HS⁎_ = emissions from the one core from treatment HS that received N and C at three times the concentration compared to the single cores in treatment ED in kg N or C ha^− 1^ h^− 1^; V_HS_ = emissions from the whole vessel of treatment HS in kg N or C ha^− 1^ h^− 1^; V_C_ = emissions from the whole vessel of the Control treatment in kg N or C ha^− 1^ h^− 1^.

### 2.4 Isotopic N_2_O

Gas sampling times for ^15^N analysis were pre-determined based on data from previous experiments (data not shown). Samples were taken just before (0 h) and 4 h after amendment, then every 24 h for the first week, followed by a final sample at day 11. This sampling strategy covered changes in isotopic signature before amendment, as well as during the main period of NO and N_2_O fluxes, and after emissions returned to background levels. Samples were taken from the outlet line of each vessel using 12 ml exetainers (Labco) which had previously been flushed with He and evacuated. ^15^N-enrichment of N_2_O was measured using a TG2 trace gas analyser (Europa Scientific, now Sercon, Crewe, UK) and Gilson autosampler, interfaced to a Sercon 20-22 isotope ratio mass spectrometer (IRMS). Solutions of 6.6 and 2.9 at% ammonium sulphate ((NH_4_)_2_SO_4_) were prepared and used to generate 6.6 and 2.9 at% N_2_O (Laughlin et al., 1997) which were used as reference and quality control standards.

The process leading to the formation of the measured N_2_O, i.e. whether it is produced by nitrification or denitrification, can be determined by calculating how much of the N_2_O derived from NO_3_^−^ as the parent molecule. When ^15^N labelled NO_3_^−^ was added, it was assumed that it completely mixed with the native soil NO_3_^−^ pool to form a single uniformly labelled NO_3_^−^ pool. The ^15^N content of the N_2_O was calculated from either ^45^R or ^46^R, with ^45^R being the ratio of the ion currents (*I*) for mass 45/44 (^45^R = ^45^*I* / ^44^*I*) and ^46^R for mass 46/44 (^46^R = ^46^*I* / ^44^*I*). If the ^15^N contents of the measured N_2_O calculated from either ^45^R or ^46^R are equal, then the distribution of the ^15^N atoms in the N_2_O molecules is random, and therefore the N_2_O originated from a single uniformly labelled NO_3_^−^ pool (Stevens et al., 1997, Stevens and Laughlin, 1998). When the NO_3_^−^ pool is labelled and the N_2_O flux is greater than the IRMS method detection limit (2 ppm) calculations of the fraction of N_2_O that derived from the denitrifying pool (*d*′_D_) can be performed. The sources of N_2_O were apportioned into *d*′_D_ and the fraction derived from the pool or pools at natural abundance *d*′_N_ = (1 − *d*′_D_) and were calculated as described in Arah (1997).

To determine the source of the measured N_2_O, i.e. how much of it was derived from the amendment (N_2_O_N_amend_) rather than the native soil N, the following equation was used for the labelled treatments (Senbayram et al., 2009):

(2) N2O_Namend=N2O_NtotalNsample15Nfert15

with N_2_O_N_total_ = total emissions of N_2_O from the soil; ^15^N_*sample*_ = ^15^N at% excess of the emitted N_2_O (^15^N at% of the measured sample minus 0.366, with 0.366 being the mean natural ^15^N abundance of background N_2_O obtained in our experiment); ^15^N_*fert*_ = ^15^N at% excess of the applied amendment solution.

### 2.5 Soil analyses

Soil samples were taken at the beginning and end of the incubation to determine the initial and final moisture contents and the NH_4_^+^ and total oxidised N (TOxN: NO_3_^−^ + NO_2_^−^) concentrations. Nitrite (NO_2_^−^) is generally thought to accumulate very rarely in nature, and it has been shown that NO_2_^−^ is rapidly transformed in soil (Paul and Clark, 1989, Burns et al., 1995, Burns et al., 1996) and previous analyses have shown that NO_2_^−^ makes up < 0.1% of the TOxN. It was therefore assumed that NO_2_^−^ concentrations within the TOxN measurements were negligible, and TOxN is nearly exclusively made up of NO_3_^−^. For these reasons TOxN will be referred to as NO_3_^−^ from this point onward. For the final soil analyses, each core was divided in half to separate the top section from the bottom section. WFPS was calculated from soil moisture contents by drying a subsample (50 g) at 105°C overnight. Soil NH_4_^+^-N and NO_3_^−^ were analysed by automated colorimetry from 2 M KCl soil extracts using a Skalar SANPLUS Analyser (Skalar Analytical B.V., Breda, Netherlands) (Searle, 1984). ^15^N-enrichment of NO_3_^−^ in the soil solution was determined at the Thünen Institute of Climate Smart Agriculture (Brauschweig, Germany) using the bacterial denitrification method (Sigman et al., 2001) and ^15^N-N_2_O obtained was analysed using a modified GasBenchII preparation system coupled to MAT 253 isotope ratio mass spectrometer (Thermo Scientific, Bremen, Germany) according to Lewicka-Szczebak et al. (2013).

### 2.6 Statistical analysis

Statistical analysis was performed using GenStat 16th edition (VSN International Ltd.). Cumulative emissions were calculated from the area under the curve after linear interpolation between sampling points. Prior to the statistical tests the data were analysed to determine whether the conditions of normality (Kolmogorov-Smirnov test) and equality of variance (Levene test) were satisfied. Where needed to fulfill these assumptions, the data were log-transformed before analysis. Differences in total emissions between treatments for each gas measured were assessed by ANOVA at p < 0.01. Where treatment effects proved to be significant, Fisher's Least Significant Test (LSD) was used to ascertain differences between treatments.

## 3 Results

### 3.1 Gas emissions

#### 3.1.1 Per vessel

**Fig. 2:**
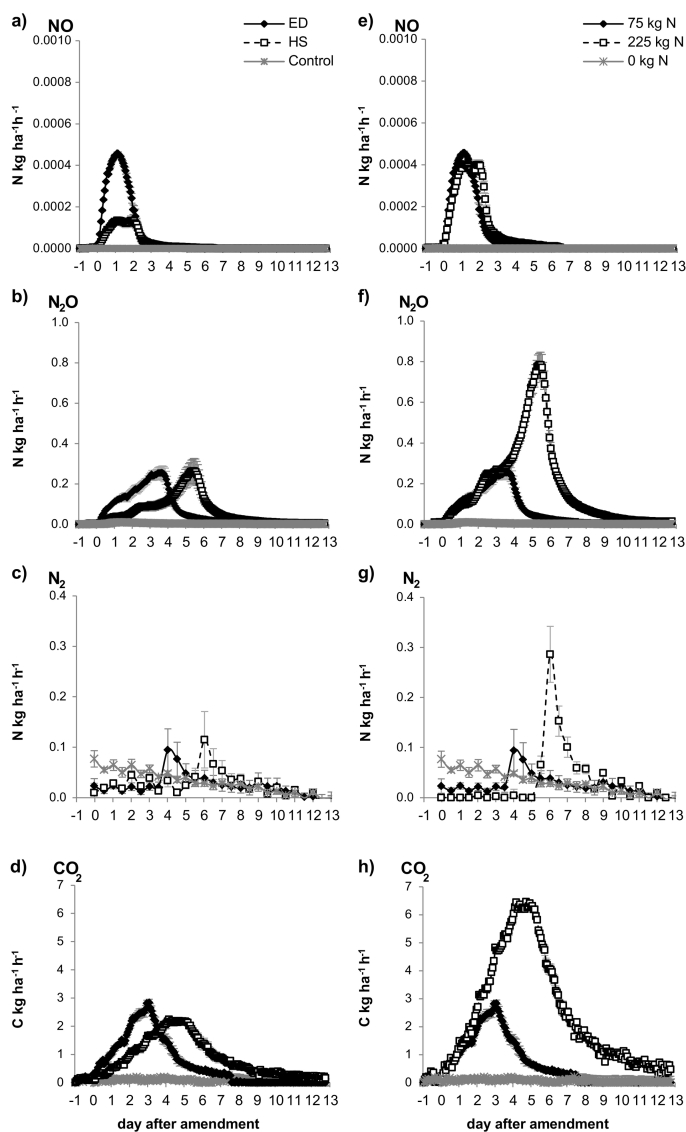
Average fluxes of NO, N_2_O, N_2_ and CO_2_ for the different treatments (n = 4). The left side (a–d) shows the gaseous emissions measured per vessel; the right side (e–h) shows emissions based on the concentration of the amendment applied to one core. (1 kg ha^− 1^ h^− 1^ = 1.74 × 10^− 5^ mg cm^− 2^ h^− 1^).

Nitric oxide (NO) emissions (Fig. 2a) increased immediately after amendment application with a peak lasting for about 2.5 days. NO emissions from the ED (equal distribution) treatment were about 4 times greater during the initial 12 h after amendment application than in the HS (Hot Spots) treatment (Table 2). Emissions from the ED treatment peaked after 26 h before decreasing again. In the HS treatment however, there was a plateau in NO emissions from about 24 to 48 h before showing the same decrease as the ED treatment. Cumulative emissions of NO (Table 3) were 2.7 times greater from the ED treatment compared to the HS treatment. Emissions of NO from the Control treatment were negligible.

**Table 3:** Cumulative emissions of NO, N_2_O and N_2_ as g N ha^− 1^ and CO_2_ as g C ha^- 1^ over the time of the respective peaks. Values 'per Vessel' are average cumulative emissions measured from the whole vessel; 'per amended core' are average cumulative emissions calculated using data for individual cores. Different letters indicate a significant difference between treatments for each measured gas (n = 4 for 'ED', 'HS', 'Control' per Vessel and '225 kg ha^- 1^'; n = 12 for '75 kg ha^- 1^' and 'Control' per amended Core; p = 0.01).

	per Vessel			per amended core		
	ED	HS	Control	75 kg ha^− 1^	225 kg ha^− 1^	Control
NO (g ha^− 1^)	18.1 ± 0.61^A^	7.0 ± 1.05^B^	0.0 ± 0.01^C^	6.0 ± 0.61^A^	7.0 ± 1.05^A^	0.0 ± 0.01^B^
N_2_O (g ha^− 1^)	19,000 ± 1700^A^	19,300 ± 2690^A^	900 ± 210^B^	6300 ± 1700^B^	18,600 ± 2690^A^	300 ± 210^C^
N_2_ (g ha^− 1^)	2900 ± 1200^A^	3300 ± 1470^A^	1500 ± 120^B^	1000 ± 1200^A^	2300 ± 1470^A^	500 ± 120^A^
CO_2_ (g ha^− 1^)	219,200 ± 13,070^A^	271,700 ± 6100^A^	21,500 ± 1380^B^	73,100 ± 13,070^B^	257,300 ± 6100^A^	7200 ± 1380^C^
Total N (g ha^− 1^)	21,900 ± 2900^A^	22,600 ± 4160^A^	2400 ± 220^B^	7300 ± 2910^A^	20,900 ± 4160^A^	800 ± 330^B^

Similar to NO emissions, N_2_O emissions increased immediately after amendment application (Fig. 2b). However, over the course of the experiment N_2_O fluxes from HS and ED showed the same shape reaching the same maximum fluxes, but at different times. The initial rate was determined over the first 24 h after amendment application and increased at a three times faster rate in the ED treatment than in the HS treatment (Table 2). In contrast to NO emissions, N_2_O emissions reached similar maximum fluxes for both treatments as well as similar cumulative emissions (Table 3). However, due to the initial slower increase in emissions the maximum N_2_O fluxes in the HS treatment were reached about 2 days later than in the ED treatment. The Control treatment only showed very small N_2_O emissions from 12 to 36 h after water addition.

Di-nitrogen gas (N_2_) emissions were initially close to baseline levels, but showed an increase 3.5 days after amendment in the ED treatment and about 5 days after amendment in the HS treatment. Similar to N_2_O emissions there was no significant difference in the maximum fluxes (Fig. 2c) or cumulative N_2_ emissions (Table 3) between the two treatments, while both were significantly higher than the Control which showed N_2_ emissions around baseline levels. The rate of increase in N_2_ concentrations was measured over 36 h following the start of the N_2_ peak (days 2.5–4.0 for the ED treatment, days 4.5–6.0 for the HS treatment). In contrast to NO and N_2_O emissions, there was no significant difference in the rates at which N_2_ emissions increased (Table 2).

Total denitrification was calculated as the sum of all N emitted (Table 3) and was not significantly different between the HS and ED treatment. However, with 9 times higher N emissions than the Control treatment, both amended treatments had a significantly higher total N loss through gaseous emissions.

Carbon dioxide (CO_2_) fluxes behaved in a similar manner to N_2_O fluxes. For both, the ED and HS treatment, CO_2_ emissions increased immediately after amendment application (Fig. 2d). In the ED treatment concentrations increased at about twice the rate of the HS treatment (Table 2) peaking after about 3 days. In the HS treatment concentrations peaked after about 4.5 days at a slightly lower maximum concentration (2.2 kg N h^− 1^) than in the ED treatment (2.8 kg N h^− 1^). With a p-value of 0.011 cumulative emissions (Table 3) were different at the 95% level. CO_2_ emissions above background levels were negligible for the Control treatment.

#### 3.1.2 Per amended area

Using Eq. (1), average emissions from cores that received 225 kg N + 1200 kg C ha^− 1^ (the one amended core from the HS treatment, HS*) could be compared to those that received N and C at a rate of 75 and 400 kg ha^− 1^ (each core in the ED treatment), and those that only received water (the two unamended cores from the HS treatment and all three cores from the Control vessels) (Fig. 2e–h, Table 3). Results show that total NO emissions were similar between the amended cores (Fig. 2e, Table 3), independent of the amount of N and C added, but significantly higher than the control cores.

Total N_2_O and CO_2_ emissions (Table 3) on the other hand were about three times higher from the core that had received 3 times the amount of N and C. Fig. 2f and h show that initial emissions up to day 2 were the same in both treatments, but while emissions decreased from the cores with the lower application rate (75 kg N) and reached background levels by day 5, emissions from the core with the higher N application (225 kg N) continued to rise, reaching their maximum at day 5 and only being reduced to background levels by day 9. N_2_ emissions from the cores receiving the lower application rate were similar to the control, but were higher from the 225 kg N amended core (Fig. 2g). Total denitrification, calculated as the sum of all emitted N gases was about three times as high from the cores with the higher amendment (225 kg N) than in the cores with the lower N and C concentration (75 kg N) (Table 3). With a p-value of 0.015 the cores with the higher rate of N and C applied show significantly higher total N emissions at the 95% confidence level.

### 3.2 ^15^N-enrichment of N_2_O and soil NO_3_^−^

The ^15^N signature of N_2_O was calculated from ^45^R or ^46^R. Results showed that for ED those values were only equal from day 1 onwards, while values for HS were only equal from day 2 onwards (data not shown). This shows that initially two pools of NO_3_^−^ (a native NO_3_^−^ pool as well as an enriched ^15^N-NO_3_^−^ pool from the amendment) existed that contributed to N_2_O emissions. Only from day 1 (ED) and 2 (HS) onwards did the N_2_O originate from a single uniformly labelled NO_3_^−^ pool (labelled amendment homogeneously mixed with native soil NO_3_^−^). Using the calculation by Arah (1997), N_2_O *d*′_D_ is the fraction of the emitted N_2_O which is derived from the ^15^N-NO_3_^−^ pool. A N_2_O *d*′_D_ value of unity (1.00) indicated that 100% of the N_2_O emitted was derived from that NO_3_^−^ pool. Values of N_2_O *d*′_D_ (Table 4) were not significantly different from unity; therefore it can be assumed that the source of the N_2_O was the uniformly mixed ^15^N-NO_3_^−^ pool.

**Table 4:** The fraction of N_2_O derived from the labelled nitrate pool (*d*′_D_).

	Time after amendment application	1d	2d	3d	4d	5d	6d	10d
ED	Mean	1.01	1.03	1.02	1.01	1.02	1.02	1.00
	S.D.	0.004	0.001	0.001	0.006	0.003	0.005	0.027
	Difference from unity (p)	NS	(0.002)	(0.003)	NS	NS	NS	NS
HS	Mean	0.85	0.94	0.97	0.99	1.00	0.98	0.84
	S.D.	0.028	0.005	0.007	0.011	0.002	0.013	0.134
	Difference from unity (p)	NS	NS	NS	NS	NS	NS	NS

NS, not significant at the p < 0.01 (two values in brackets give the p-value for those samples that were different at the 99% but not at the 99.9% confidence level).

**Fig. 3:**
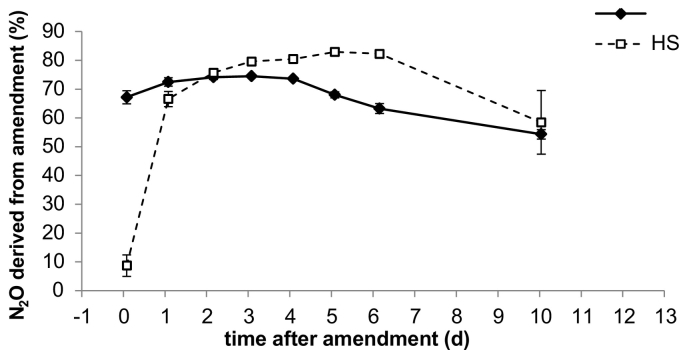
Portion of N_2_O derived from ^15^N enriched amendment in percent of total emitted N_2_O. Error bars are standard error (n = 4).

The emitted N_2_O of the labelled treatment was analysed for ^15^N enrichment. Results of this experiment are presented in Fig. 3 and show that for the ED treatment the ^15^N enrichment of N_2_O showed, that up to day 4 around 70% of the emitted N_2_O was derived from the applied amendment, with a constant decrease afterwards. If only 1 core within a vessel received amendment (HS treatment) the enrichment was initially low indicating that initially most of the N_2_O (90%) derived from the native soil NO_3_^−^, though N_2_O concentrations at this point were very low and ^15^N results should therefore be treated with caution. However, the enrichment in ^15^N of the N_2_O quickly increased within the first day with the percentage of amendment derived N_2_O reaching levels similar to those detected from the ED treatment. After this the contribution of the ^15^N enriched treatment to the total N_2_O emissions increased to around 82% only showing a decrease after day 6 to 58%. By day 10 values in HS were similar to ED, however, by this time emissions were again, very low (Fig. 2, Fig. 3).

### 3.3 Soil mineral N

**Fig. 4:**
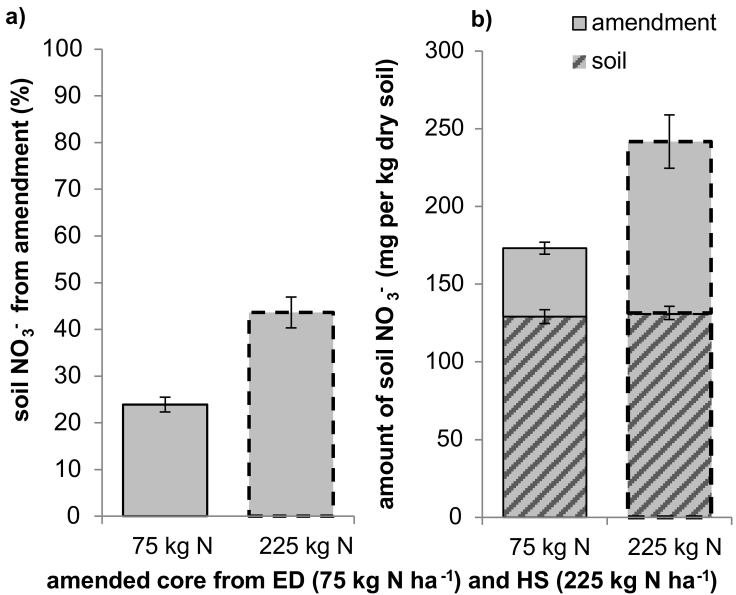
Soil NO_3_^−^ (a) ratio of amendment derived NO_3_^−^ remaining in the soil and (b) total amounts of amendment derived and native soil NO_3_^−^ in the soil at the end of the incubation.

Results of the final soil analysis are given in Table 5. Nitrate concentrations (NO_3_^−^) were only significantly different between the top and the bottom half of the cores for the control treatment. No significant difference could be detected within any of the amended treatments. Looking at the whole vessel (HS, ED, Control), there was no significant difference in the concentrations of NO_3_^−^ between the HS and ED treatments and both were similar to the Control with only the ED treatment showing higher amounts of NO_3_^−^ in the bottom half. When considering only the amended core out of each treatment, the core amended with 225 kg N ha^− 1^ from the HS treatment (column 225 kg ha^− 1^) showed significantly higher concentrations of NO_3_^−^ than the other cores both at the top as well as at the bottom of the core. The ^15^N enrichment of NO_3_^−^ was higher in the top half (1.683 ± 0.423 and 2.611 ± 0.508 at% for the 75 and 225 kg N ha^− 1^ amended cores, respectively) than in the bottom half (1.469 ± 0.327 and 2.514 ± 0.491 at% for the 75 and 225 kg N ha^− 1^ amended cores, respectively) of the cores in all amended cores. The enrichment was significantly higher in the cores receiving the higher N concentration (p < 0.01). By the end of the experiment about 45% of the soil NO_3_^−^ remaining originated from the amendment, equating to 110.3 mg N kg^− 1^ dry soil, while in the cores amended with the lower N concentration about 25% of the remaining NO_3_^−^ originated from the amendment, equating to 44.0 mg N kg^− 1^ dry soil (Fig. 4).

**Table 5:** Results of soil analysis at the end of the experiment.

		HS	ED/75 kg ha^− 1^	225 kg ha^− 1^	Control
TON mg N kg^− 1^ dry soil	Top	180.4 ± 17.90^B^	170.9 ± 15.15^B^	242.5 ± 37.96^A^	156.8 ± 1.81^B^
	Bottom	180.6 ± 15.28^BC^	175.4 ± 7.70^B^	241.0 ± 26.54^A^	156.3 ± 1.04^C^
NH_4_ mg N kg^− 1^ dry soil	Top	7.7 ± 0.40*^A^	5.6 ± 0.11*^B^	7.0 ± 0.63*^A^	7.0 ± 0.20*^A^
	Bottom	16.1 ± 2.37*^B^	12.4 ± 0.90*^B^	25.1 ± 4.51*^A^	10.1 ± 0.43*^C^
WFPS %	Top	81.57 ± 0.255*			
	Bottom	73.64 ± 0.228*			

Total amounts measured for NH_3_^−^ and NH_4_^+^. ‘HS’ = average values for 12 cores (4 amended with 225 kg N ha^− 1^, 8 unamended) from vessels of treatment HS; ‘ED/75 kg ha^− 1^’ = average values for 12 cores (12 amended with 75 kg N ha^− 1^) of treatment ED which is equivalent to the average of all cores amended with 75 kg N ha^− 1^; ‘225 kg ha^− 1^’ = average values for the 4 cores of treatment HS that received 225 kg N ha^− 1^. ‘Control’ = average of 12 cores from the Control treatment only receiving water. Different letters indicate a significant difference between treatments for each layer (Top or Bottom); * indicates significant difference between the top and bottom layers within a single grouping. (n = 12 for ‘HS’, ‘ED/75 kg ha^− 1^’ and ‘Control’, n = 4 for ‘225 kg ha^− 1^’, p = 0.01).

The soil NH_4_^+^-N concentrations were lower than NO_3_^−^ concentrations at the end of the incubation in all treatments with significantly higher values in the bottom section of the core. Looking at the whole vessel as well as individual amended cores: the vessel/core receiving 75 kg N ha^− 1^ (ED treatment) showed significantly lower amounts of NH_4_^+^ (both in the top and bottom half of the core) than the vessel (and also the 225 kg N ha^− 1^ amended core), from the HS treatment. The Control treatment showed NH_4_^+^ amounts similar to the HS treatment at the top and significantly lower amounts at the bottom of the cores.

Soil moisture was 85% WFPS at the start of the incubation and remained similar between all cores irrespective of treatments.

## 4 Discussion

### 4.1 Gaseous emissions

Only negligible gaseous emissions were detected in the control treatment. It can therefore be assumed that N_2_O emissions in the HS and ED treatments result almost exclusively from the amendments, which was confirmed by ^15^N analysis (see below). Overall, total emissions of N_2_O, N_2_ and CO_2_ were not significantly different between the HS and ED treatment, meaning that the one amended core in the HS treatment produced three times the amount of gases than one core within the ED treatment. This indicates that the emission of those gases is related to the amount of applied NO_3_^−^ and C, i.e. NO_3_^−^ and C being the factors limiting denitrification activity, rather than the soil area (and mass) that receives the amendment. Therefore, three times more N_2_O, N_2_ and CO_2_ were produced when three times the amount of KNO_3_ was applied. A similar effect has been observed by Wang et al. (2013) who found increasing N_2_O, N_2_ and CO_2_ emissions with increasing initial NO_3_^−^ concentrations.

Though total emissions were similar, the peak of N_2_O and N_2_ fluxes was delayed by about 2 days in the HS treatment. There was no leaching in this experiment, therefore this delay implies that the applied nutrients remained in the soil for a longer period in the HS compared to the ED treatment, where the transformation products in the form of N_2_O were detected and increased immediately after nutrient application. In contrast to this, NO emissions were three times lower in the HS treatment as compared to the ED treatment meaning that emissions from each amended core were the same, independently of the amount of KNO_3_ applied. This suggests that NO emissions were related to the area (or soil volume) that received the amendment and not the amount of applied nutrients. NO emissions are therefore not a good indicator of hot-spot activity.

### 4.2 Denitrification reactions

In the ED treatment the amendment solution was spread over all three cores supplying a three times larger microbial community with the nutrients than in the HS treatment. The lower amounts of NO emitted from the HS treatment can be explained by both, a larger microbial community accessing the supplied NO_3_^−^ substrate in the ED treatment, as well as a delay in the production of NO reductase (Nor) – the enzyme responsible for reducing NO to N_2_O. In the HS treatment a smaller microbial community was supplied with the NO_3_^−^ substrate and less NO was produced than by the larger community in the ED treatment which resulted in smaller initial emission rates. The microbial community using the NO_3_^−^ substrate could grow and was therefore able to reduce more NO_3_^−^ to NO. However, by the time the community was increasing NO production, it had also had time to develop the ability to further reduce the NO to N_2_O. The consumption of NO then resulted in a plateau in NO emissions in the HS treatment after just over 24 h.

A similar pattern for NO emissions was also found by Wang et al. (2013). While they found that cumulative NO emissions increased with initial NO_3_^−^ concentrations when those were below 50 mg N kg^− 1^ dry soil, they found no difference in NO emissions at higher concentrations. Similarly, Shannon et al. (2011) found no difference in the activity of Nor in an experiment where they inoculated *Pseudomonas mandelii* into anoxic soil with glucose (500 mg C kg^− 1^ dry soil) and NO_3_^−^ at concentrations ranging from 0 to 500 mg N kg^− 1^ dry soil. In addition, it has been shown that the production of Nor is delayed by 24 to 48 h following the onset of anaerobic conditions (Saggar et al., 2013). However, NO emissions are not solely dependent on the NO_3_^−^ concentration but also on the soil water content, pH, the soil temperature and the ambient NO concentration (Ludwig et al., 2001, Obia et al., 2015).

In contrast to NO emissions, N_2_O emissions were similar between the HS and ED treatments, but calculating the gaseous N emissions per amended core confirmed a higher amount of N_2_O emitted from the cores receiving the higher concentration of KNO_3_ and C (225 kg N and 1200 kg C ha^− 1^), meaning that total emissions were related to the amount of N and C applied and independent of the area they were applied to. During denitrification N_2_O is the product of NO reduction. The low amounts of detected NO are explained by NO being reduced to N_2_O before it can reach the soil surface and be measured. Following the denitrification process, N_2_O should be further reduced to N_2_. Although N_2_ concentrations were elevated in the core with the higher concentrated amendment, concentrations were low and the difference to the cores receiving the lower N amendment was not significant.

This result can be explained by the metabolism of the denitrifying microbial community. Because of NO being membrane-labile and highly toxic, most bacteria, including all denitrifiers, synthesise the Nor enzyme to reduce NO to N_2_O to avoid poisoning. However, many denitrifiers lack one or more of the other enzymes to catalyse all reduction steps during denitrification (Saggar et al., 2013). This very often is the N_2_O reductase (Nos) which reduces N_2_O further to N_2_. Additionally, energy yields from denitrification reactions lessen in order of their sequence, with the reduction of NO to N_2_O being more energetically favourable than the reduction of N_2_O to N_2_ (Koike and Hattori, 1975, Saggar et al., 2013). The relatively high amounts of N_2_O being produced while amounts of N_2_ detected in this experiment were very low can be explained by a combination of the factors mentioned above, which promote an accumulation of N_2_O. Additionally, NO_3_^−^ was present in abundance and denitrification requires available C, which was also applied, but might have become limiting before the NO_3_^−^ was used up and therefore not making the microorganisms perform the last, less energetically favourable step of reducing N_2_O to N_2_.

Carbon dioxide emissions are a measure of biological activity and are often used to indicate microbial activity or respiration (Parkin et al., 1996). Denitrification requires an electron donor such as C. In this experiment glucose-C was applied resulting in the production of CO_2_. The measured CO_2_ concentrations increased similarly to the N_2_O emissions, peaking just before the maximum N_2_O emissions were measured. The simultaneous occurrence of peak CO_2_ and N_2_O fluxes may indicate both denitrifying and other heterotrophic microbes being active at the time (Tiedje, 1988).

### 4.3 Molar ratios of denitrification gases

Ratios of NO:N_2_O as well as N_2_O:N_2_ have been used as indicators of the relative contributions of nitrification and denitrification to the detected NO and N_2_O emissions. For the ED and HS treatment the molar NO:N_2_O emission rates in this experiment decreased from 0.0046 to 0.0002 during the first 5 days due to a decrease in NO emissions and an increase in N_2_O emissions. With decreasing N_2_O emissions those ratios increased again to 0.0016 by day 7 after which NO emissions were below the detection limit. In the Control, ratios decreased similarly until day 1.5 but then showed a gradual increase to 0.012 until day 7. Ratios of total, cumulative emissions were below 0.001 for all treatments irrespective of whether an amendment was applied and how (i.e. as a hot-spot (HS) or equally distributed (ED), as a high (225 kg ha^− 1^) or low (75 kg ha^− 1^) concentration, or without nutrient addition (Control)).

Values < 0.01 have been associated with denitrification and restricted aeration (Skiba et al., 1992) and while our results fit with this assumption it should be noted that other studies clearly showed that using the NO:N_2_O ratio as an indicator to judge whether nitrification (NO:N_2_O > 1) or denitrification (NO:N_2_O < 1) was the dominating source process must be reconsidered (del Prado et al., 2006, Scheer et al., 2009, Wang et al., 2011, Wang et al., 2013). The N_2_:N_2_O ratios peaked with the N_2_ peak of the respective treatment. The largest ratios of N_2_:N_2_O are expected if available C is high and the denitrification reactions are followed all the way to N_2_, whereas if NO_3_^−^ concentrations are high, but available C is low, the reduction of N_2_O to N_2_ is inhibited and N_2_O may be the sole end product, resulting in a low N_2_:N_2_O ratio (Wang et al., 2011). Ratios of cumulative emissions were around 0.1 for the amended treatments (HS, ED, 75 kg ha^− 1^, 225 kg ha^− 1^) and 1 for the Control treatment. Decreasing ratios of N_2_:N_2_O after day 4 in ED and after day 6 in HS indicate C limitation in this experiment. However, great ranges of ratios have been reported in the literature from < 1 to 200 indicating that those ratios can vary significantly depending on soil NO_3_^−^, C availability, redox potential, soil properties and denitrifier activity (Wang et al., 2013).

### 4.4 ^15^N-N_2_O

^15^N analysis was used to determine whether the native soil NO_3_^−^ or the NO_3_^−^ added with the amendment was the source of the emitted N_2_O. Results showed that emissions measured in the ED treatment were mainly from the added NO_3_^−^ throughout the whole incubation period. In the HS treatment, however, a low ^15^N enrichment of the measured N_2_O after 4 h indicates that during the first few hours most of the emitted N_2_O was from the native soil NO_3_^−^-pool. As the production of N_2_O is low at this stage, the N_2_O produced from the non-amended cores is likely to mask the effect of the amendment on N_2_O production. While the microbial communities receiving nutrient amendment are expected to be stimulated to the same extent, in the HS treatment only one third of the soil/microbial community received nutrient amendment. The lower percentage of amendment-derived N_2_O 4 h after N application in the HS treatment may be explained by this smaller volume of soil/the microbial community receiving the enriched amendment. At this stage the two cores that only received water within this treatment were producing N_2_O from native soil N sources, like the Control treatment. The higher ratio of amendment-derived N_2_O in the HS treatment possibly results from the relative enhanced accessibility of amendment within a small core volume replacing the use of native soil N which might be harder to access for the microbial community.

Fig. 4b showed that at the end of the experiment in both treatments about 130 mg NO_3_^−^-N kg^− 1^ remained which was not derived from the amendment. This large total amount of NO_3_^−^ at the end of the experiment indicates that denitrification reactions might have stopped due to a lack of available C.

## 5 Conclusions

The results of our study showed that under the given conditions NO emissions were proportional to surface area, while N_2_O emissions were proportional to nutrient concentration.

Results of this experiment showed that applying nutrients in a localised manner reduced the rate of NO emissions, a gas of environmental concern. At the same time it delayed gaseous emissions of N_2_O, resulting in a longer residence time of the parent compound in the soil.

This study therefore showed that emissions of different gases are not influenced by the same factors in the same way. The amount of NO emissions depend on the area/soil volume that received KNO_3_ and C fertiliser, while the scale of N_2_O and N_2_ emissions depends on the amount of the applied KNO_3_ and available C.

Our results indicate that, under conditions promoting denitrification, the tendency for higher activity at nutrient hot-spots is greater for N_2_O and N_2_ emissions. Due to the relatively lower amounts of emitted NO, the contribution of this gas on the total gaseous emissions of N was negligible. However, with mitigation strategies reducing emissions of N_2_O, NO will become of more interest in the future and different factors influencing its emission will need to be considered and incorporated into mitigation strategies.

This study was performed under highly controlled conditions necessary to investigate effects of single factors. However, due to these conditions it cannot be scaled up to the field scale. Further experiments are needed to expand our knowledge about conditions affecting emissions. While this study did not include mechanistic investigations, future studies should be performed to include analyses such as methods to determine denitrification kinetics. It is possible that DNRA (or nitrate ammonification) contributed to these emissions, although several studies have demonstrated this process to be low under high nitrate conditions, such as in our experiment (e.g. Rütting et al. (2011), van den Berg et al. (2015)).

Additionally, this experiment was performed to investigate soil effects only, however, in future experiments, when introducing plants to the system, it is expected that this delay in NO_3_^−^ reduction will give those plants more time to take up the NO_3_^−^, therefore reducing the amount of NO_3_^−^ in the soil. Decreasing NO_3_^−^ as an energy source for denitrifiers can not only result in lower N_2_O emissions due to a lower availability of substrate, but also due to driving those organisms to perform the subsequent and less energetically favourable step of denitrification, i.e. using N_2_O to produce N_2_ and hence lowering GHG emissions even further.
